# P-Loop Residues Critical for Selectivity in K^+^ Channels Fail to Confer Selectivity to Rabbit HCN4 Channels

**DOI:** 10.1371/journal.pone.0007712

**Published:** 2009-11-05

**Authors:** Nazzareno D'Avanzo, Roman Pekhletski, Peter H. Backx

**Affiliations:** Departments of Physiology and Medicine, Heart and Stroke/Richard Lewar Centre, University of Toronto, Toronto, Ontario, Canada; University of Cincinnati, United States of America

## Abstract

HCN channels are thought to be structurally similar to K_v_ channels, but show much lower selectivity for K^+^. The ∼3.3 Å selectivity filter of K^+^ channels is formed by the pore-lining sequence XT(V/I)GYG, with X usually T, and is held stable by key residues in the P-loop. Differences in the P-loop sequence of HCN channels (eg. the pore-lining sequence L_478_C_479_IGYG) suggest these residues could account for differences in selectivity between these channel families. Despite being expressed, L478T/C479T HCN4 channels did not produce current. Since threonine in the second position is highly conserved in K^+^ channels, we also studied C479T channels. Based on permeability ratios (P_X_/P_K_), C479T HCN4 channels (K^+^(1)>Rb^+^(0.85)>Cs^+^(0.59)>Li^+^(0.50)≥Na^+^(0.49)) were less selective than WT rabbit HCN4 (K^+^(1)>Rb^+^(0.48)>Cs^+^(0.31)≥Na^+^(0.29)>Li^+^(0.03)), indicating that the TIGYG sequence is insufficient to confer K^+^ selectivity to HCN channels. C479T HCN4 channels had an increased permeability to large organic cations than WT HCN4 channels, as well as increased unitary K^+^ conductance, and altered channel gating. Collectively, these results suggest that HCN4 channels have larger pores than K^+^ channels and replacement of the cysteine at position 479 with threonine further increases pore size. Furthermore, selected mutations in other regions linked previously to pore stability in K^+^ channels (ie. S475D, S475E and F471W/K472W) were also unable to confer K^+^ selectivity to C479T HCN4 channels. Our findings establish the presence of the TIGYG pore-lining sequence does not confer K^+^ selectivity to rabbit HCN4 channels, and suggests that differences in selectivity of HCN4 versus K^+^ channels originate from differences outside the P-loop region.

## Introduction

Hyperpolarization-activated cyclic-nucleotide gated (HCN) channels are expressed distributed in several excitable tissues including neurons and cardiomyocytes [1] where they contribute to pacemaker electrical activity [2], [3]. Four mammalian isoforms have been cloned (HCN1 to 4), each with different activation and deactivation kinetics as well as sensitivity of activation properties to cAMP [4]–[6]. Although, HCN channels share many sequence and structural similarities with voltage-gated K^+^ (K_v_) channels, they possess unique selectivity, permeation, and gating properties. Specifically, in contrast to other voltage-gated channels, HCN channels activate slowly in response to membrane hyperpolarization despite similar voltage-sensor movement [7], [8]. HCN channels also have relatively high permeability to Na^+^ ions compared to K^+^ channels [9]–[11]. While the unique gating properties of HCN channels has been the focus of several recent studies [7], [8], [12], the molecular basis of differences in selectivity between HCN and K^+^ currently remains unknown.

**Figure 1:**
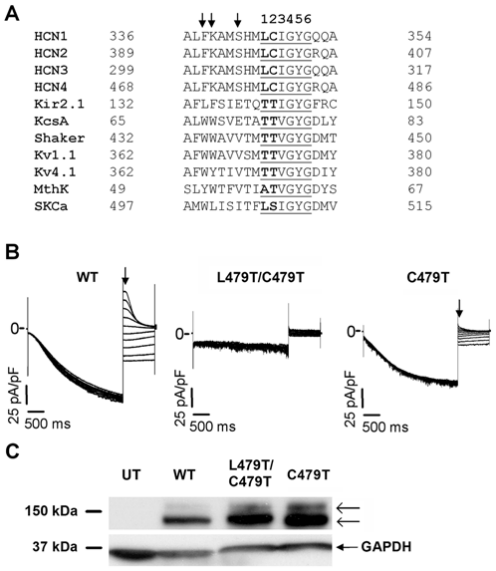
Ion selectivity in WT and C479T HCN4 channels. **(A)** Sequence alignment of mammalian HCN channels, and various K^+^ selective channels shows all mammalian HCN channels have a leucine and cysteine residue in place of the two threonines typically (though not always) present in the underlined K^+^ channel ‘selectivity sequence’. These residues (L478 and C479 in HCN4) were mutated to threonine to create a pore-lining sequence resembling that of a K^+^ selective channel. **(B)** Sample traces of WT, L478/C479T, and C479T HCN4 currents recorded in a 5 mM K^+^/135 mM Na^+^ bath solution, elicited by a 3 s prepulse to −130 mV from a holding potential of 0 mV, followed by a 1 s test pulse between +20 to −70 mV (ΔV = 10 mV). **(C)** Western blots performed from whole-cell lysates of untransfected (UT) CHO-K1 cells, or cells transfected with WT, L478/C479T or C479T constructs, with GAPDH used as a loading control. WT and mutant channels are highly expressed in both *N*-glycosylated and core bands (←), indicating the absence of L478/C479T currents in expressed cells is not due to mis-trafficking or low protein production.

One potential source of differences in selectivity between K^+^ channels and HCN channels is the selectivity sequence of the P-loop, as previously suggested [1], [13], [14]. Heteromeric K^+^ channels have a pore-lining sequence of TT(V/I)GYG (Figure 1A) which forms 4 equally spaced ion-binding sites [15], [16] that confer K^+^ selectivity to these channels [17]. By contrast, HCN channels have a pore-lining sequence of LCIGYG (Figure 1A) and have a 20–fold lower selectivity for K^+^ over Na^+^ ions compared to K^+^ channels [11], [18], [19]. Consistent with P-loop differences underlying the distinct ion selectivity between HCN and K^+^ channels, T75C mutant *KcsA* channels show marked reductions in K^+^ conductance and altered ion binding [20], while T441S and T442S/G mutant *Shaker* channels have increased conductance of Rb^+^ and NH_4_^+^ ions [21], [22].

Other regions of the P-loop sequence could also be responsible for selectivity differences between HCN and K^+^ channels. For example, two pore-helix Trp residues along with the Tyr side-chain of the GYG region stack and form a hydrophobic cuff around the selectivity filter in *KcsA* channels that confers pore rigidity and holds the pore open at its diameter [15]. Disruption of this aromatic sheet in *Shaker* channels, via a W434F mutation, permits substantial Na^+^ current in the absence of K^+^ ions [23], [24]. Unlike K^+^ channels, HCN channels have Phe (F471) and Lys (K472) residues in place of Trp residues at the equivalent positions (Figure 1A). In addition, stabilization of the tertiary structure of the selectivity filter in inward rectifier K^+^ channels is also linked to salt bridge formed between E138 and R148 using Kir2.1 nomenclature [25]. Moreover, disruption of the equivalent “bow string” salt bridges of Kir3.1/Kir3.4 increases the pore diameter, increases pore flexibility, and reduces channel selectivity [26]. Interestingly, HCN channels also have an Arg residue (R484) on the extracellular side of the selectivity filter near the one found in Kir2.1 channels, however, have a Ser (S475) at the equivalent location for the negative counter ion (E138 of Kir2.1 channels) required to form a salt bridge. Thus, HCN channels may lack the molecular components required to stabilize pore structure and rigidity. Consistent with this conjecture, molecular models of spHCN1 and HCN2 channels show that HCN channels may have fewer stabilizing interactions between the selectivity filter and the P-loop α-helix than observed in K^+^ channels [27] suggesting greater flexibility within the HCN pore.

To identify a molecular basis for differences between ion selectivity in HCN channels versus K^+^ channels, we examined the role of P-loop residues known to be critical for selectivity in K^+^ channels but naturally substituted in HCN channels. To this end, we replaced the selectivity filter leucine and cysteine residues of HCN4 channels at positions 478 and 479 respectively with threonines. Additionally, strategic site-directed mutant HCN4 channels were generated (F471W/K472W, S475D, and S475E channels in a C479T background) in an attempt to introduce stabilizing interactions that may confer K^+^ selectivity to HCN channels. Our data shows C479T channels were less, not more, selective for K^+^ ions over other monovalent cations than WT HCN4, and S475D/C479T HCN4 channels did not improve ion selectivity. L478T/C479T, F471W/K472W/C479T and S475E/C479T HCN4 channels did not produce any measurable currents despite high protein expression. The permeability sequence pattern of HCN4 and C479T HCN4 channels to alkali metals and organic cations indicates that HCN4 channels have larger pores than typical K^+^ channels, which may contribute to their reduced ionic selectivity. The C479T mutation further increases the minimum pore diameter in HCN4 channels, consequently further reducing ionic selectivity. Thus, the presence of the K^+^ channel selectivity filter sequence, TIGYG, does not confer selectivity to HCN4 channels suggesting that HCN channels may be more structurally divergent from K^+^ than anticipated. The implications of these larger pores on HCN channel conductance and gating are also discussed below.

## Materials and Methods

### Mutagenesis

Rabbit HCN4 cDNA (GenBank accession number AB022927) was kindly provided by Dr. H. Ohmori (Kyoto University, Kyoto, Japan) and subcloned into the pBiG vector (Clontech Inc, Palo Alto, CA) in which we replaced the *lacZ* reporter with enhanced green fluorescence protein (Clontech Inc, Palo Alto, CA). Thus, expression of HCN4 and EGFP was under control of the tetracycline transactivator (tTA). Kozak sequences were also inserted into pBiG to improve expression. Site-directed mutant HCN4 channels were generated by PCR-mutagenesis using *Pfu* DNA Polymerase (Promega, Madison, WI), and sequenced to ensure fidelity.

### Expression and Electrophysiological Recordings of Rabbit HCN4 Channels in Chinese Hamster Ovary (CHO)-K1 Cells

CHO-K1 cells (ATCC, Manassas, VA) were cultured at 37°C, 5% CO_2_ in F12 media (SIGMA), supplemented with 10% FBS and 1% penicillin/streptomycin (Gibco BRL) and transfected with 2 µg of WT or mutant HCN4 cDNA plus 1 µg of tTA cDNA using 3 µL of Lipofectamine 2000 (Gibco BRL, Carlsbad, CA) following the supplied protocol. Transfected cells were incubated in supplemented culture media (see above) for 24-hours prior to the electrophysiological recordings.

Cells expressing GFP were optically chosen for whole-cell recordings 24–48 hours after transfection. The pipette solution contained (in mM): 140 K^+^ (Cl^−^ and OH^−^), 1 EGTA, and 10 HEPES (pH = 7.4 with KOH). Two types of external solutions were used. One contained (in mM) 5 K^+^, 135 X^+^ (X = K, Na, Rb, Li, Cs, NH_4_, MA, DMA, TMA, or TEA) and the other contained 140 Na^+^. Each external solution also contained (in mM) 5 HEPES, 1 MgCl_2_, 1.8 CaCl_2_ and were pH adjusted to 7.4 with the appropriate hydroxide. TW140F-4 patch pipettes (World Precision Instruments, Florida) were pulled and fire-polished to give resistances of 2–3 MΩ. All recordings were performed after 2 mins of dialyzing the internal solution following membrane rupture in order to avoid issues of current rundown. In all recordings, each test pulse was followed by a 17–24 s interpulse period at the holding potential to ensure complete channel deactivation. Currents were recorded at room temperature at 20 kHz using an Axopatch 200B amplifier, (Axon Instruments) and filtered at 2 kHz. Capacitance and series resistance were electronically compensated by >75%.

Single-channel recordings were done using the cell-attached patch clamp technique. Bath solutions contained (mM): 140 K^+^ (Cl^−^ and OH^−^), 5 HEPES, 1 MgCl_2_, and 1.8 CaCl_2_ (pH = 7.4, with KOH). Pipette solutions contained (in mM): 140 K^+^ (Cl^−^ and OH^−^), 5 HEPES, 1 MgCl_2_, and 1 EGTA (pH = 7.4 with KOH). TW140F-4 patch pipettes (World Precision Instruments, Florida) were pulled and fire-polished to give resistances of >10 MΩ for single channel recordings. Pipettes were coated with Sylgard (Dow Corning Corporation, Michigan) to reduce their capacitance. Data were acquired using Axopatch 200 A amplifier, collected at 5 kHz by Clampex 6 (pCLAMP, Axon Instruments) with a 1 kHz low-pass Bessel filter. Currents were further filtered offline at 0.4 kHz and analyzed using Clampfit 9 (pCLAMP, Axon Instruments, Foster City, CA).

Reversal potentials (E_rev_) of HCN4 currents were determined from leak subtracted instantaneous currents elicited by a 3 s prepulse to −130 mV, followed by a 1 s test pulse to depolarized potentials between +20 mV and −70 mV in 10 mV increments from a holding potential of 0 mV. Leak was determined from linear fits of residual currents at the end of 10 ms capacitance recordings. Permeability ratios (P_X_/P_K_) were determined from the E_rev_ measurements by using the Goldman-Hodgkin-Katz equation
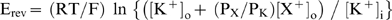
(1)where R, T and F have their usual meanings.

Activation kinetics were estimated from currents elicited by test pulses between −140 to −70 mV (ΔV = 10 mV) with the duration increasing by 850 ms with each +10 mV increment in voltage. Time constants of activation (τ_act_) and deactivation (τ_deact_) of HCN4 currents were determined by fitting currents with a mono-exponential function after the initial lag. Steady-state activation currents in the presence of 5 mM K^+^/135 mM Na^+^ or 140 mM K^+^ were measured from tail currents at +30 mV following prepulses ranging from −150 to −60 mV in 10 mV increments, which increased in duration by 850 ms with each more depolarized potential. I/I_max_ was plotted as a function of test potential and values were fit with a Boltzmann function
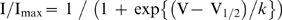
(2)to determine the midpoint of activation (V_1/2_) and slope factor (*k*).

### Excluded Field Theory

To obtain estimates of the pore diameter of WT and C479T HCN4 channels we recorded currents in the presence of the organic cations NH_4_^+^, MA^+^, DMA^+^, TMA^+^, and TEA^+^ as originally described in Dwyer *et al.* (1980) [28]. These recordings satisfy the conditions and assumptions for the application of Excluded Field Theory (EFT), which relates P_X_/P_K_ to the pore radius, R_p_, and the crystal radius of ion X^+^, r_c,x_, by the equation [28]

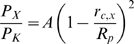
(3)


Thus, the pore diameter (2R_p_) can be determined by fitting our measured P_X_/P_K_ data and published values for r_c,x_.

### Western Blots

Western blot analysis was performed using protein extracts from untransfected CHO-K1 cells, and CHO-K1 cells transfected with either WT or mutant HCN4 channels. Lysis buffer contained 50 mM TRIS (pH = 8.8), 250 mM NaCl, 5 mM EDTA, 10% glycerol, 0.1 mM Na_3_VO_4_, 0.1% NP-40, and 1.2% Triton X-114, supplemented with a protease inhibitor cocktail (“Complete”; Roche Diagnostics, Germany). Proteins were separated by an 8% SDS-PAGE and transferred to polyvinylidene fluoride (PVDF) membranes (Roche, Germany). Blocking was achieved by incubating the membrane with PBS supplemented with 0.1% Tween-20 (PBST), and 5% powdered skimmed milk overnight at 4°C. Membranes were blotted with the polyclonal anti-HCN4 antibodies (Alomone Labs, Israel) (1∶500 dilution) in PBST supplemented with 5% BSA. Secondary anti-rabbit antibodies coupled to horseradish peroxidase (HRP) (Bio-Rad, Mississauga, ON) were used at a 1∶5000 dilution and the bands visualized by incubation in ECL Western blotting detection reagents (Amersham Biosciences, Italy). Blotting for GAPDH was used as a loading control.

### Analysis and Statistics

Recordings were analyzed using Clampfit 8.0 (Axon Laboratories, Foster City, CA) with all other data analysis, plotting, and curve fitting performed with Origin 6.0 (Microcal Software Inc., Northampton, MA). Student's t-tests were used for comparisons carried out between mutant versus wild-type channels. P-values were considered significant if they were <0.05, unless otherwise stated.

## Results

Variations in the pore-lining sequences have been suggested to contribute to differences in ion selectivity between of K^+^ channels (TT(I/V)GYG) and HCN4 channels (LCIGYG) (Figure 1A) [1], [13], [14]. Therefore, we replaced L478 and C479 residues in the HCN4 channel pore with threonines. Unfortunately, currents were not observed when L478T/C479T HCN4 channels were expressed in CHO-K1 cells whether the external solution contained 5 mM K^+^ plus 135 mM Na^+^ (Figure 1B) or 140 mM K^+^ (data not shown). To determine whether L478T/C479T channel proteins were in fact expressed, Western blots were performed on whole-cell lysates. We observed high expression levels for both WT and L478T/C479T HCN4 channels (Figure 1C) with *N*-glycosylated and unglycosylated bands being clearly evident. These results suggest that the absence of current in L478T/C479T channels results from non-functioning protein in the plasma membrane, since glycosylation is required for HCN protein trafficking [29]. Alternatively, the absence of current in these channels may originate from large negative shifts in their voltage-dependence for activation. (i.e. to voltages below −130 mV).

**Figure 2:**
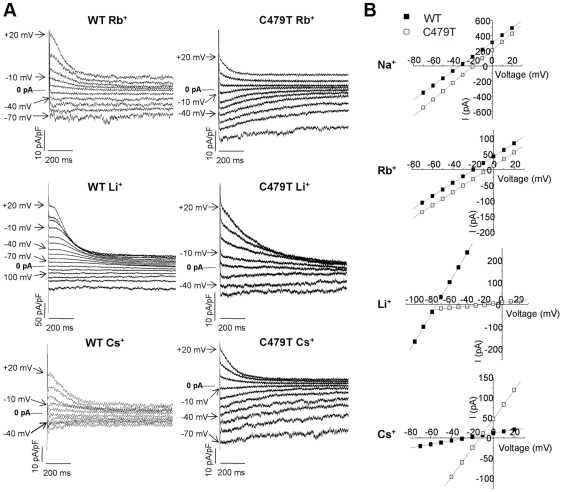
E_rev_'s determined from the instantaneous I–V relationship. **(A)** Sample tail currents elicited following an activating “pre-pulse” to −130 mV as shown in Figure 1B for experiments performed in 5 mM K^+^ and 135 mM X^+^ (Li^+^, Rb^+^, Cs^+^) extracellular bath solution. **(B)** Representative instantaneous I–V relationships for recordings performed in 5 mM K^+^ and 135 mM X^+^ (Na^+^, Li^+^, Rb^+^, Cs^+^) bath solution WT (▪) and C479T (□) channels. E_rev_'s were determined from the linear fits of the I–V relationship for each cell, and were then used to calculate the P_X_/P_K_ using the GHK equation and averaged as presented in Table 1.

Since the threonine in the selectivity sequence at position 2 is highly conserved (Figure 1A), and forms the fourth ion binding site in K^+^ channels [15], [16], we went on to examine the biophysical properties of C479T mutant HCN4 channels. To assess the selectivity properties of C479T channels, E_rev_ was determined from linear fits of leak subtracted instantaneous tail currents (Figure 2). Using the Goldman-Hodgkin-Katz equation (Eq. 1), we found that selectivity of ion “X” in WT HCN4 channels, quantified as P_X_/P_K_, was K^+^ (1)>Rb^+^ (0.48)>Cs^+^ (0.31)≥Na^+^ (0.29)>Li^+^ (0.03) (Table 1). This is consistent with a type IV or V Eisenman sequence [30], [31] indicating a mild anionic field strength in the pore of HCN4 channels. In C479T channels, the selectivity sequence was altered to K^+^ (1)>Rb^+^ (0.85)>Cs^+^ (0.59)>Li^+^ (0.50)≥Na^+^ (0.49) with P_X_/P_K_ increasing (P<0.05) for all test ions (Table 1) particularly for Li^+^ which has the largest hydrated radius. This sequence of permeabilities is in line with a “Li anomaly” Eisenman sequence [31] still suggestive of type IV mild anionic field strength in the mutant pore. These data establish that the TIGYG pore sequence found in most selective K^+^ channels is insufficient to confer K^+^ selectivity to HCN4 channels.

**Table 1:** E_rev_ and P_X_/P_K_ estimates in WT and C479T HCN4 channels.

	Conditions	WT HCN4	C479T HCN4
**E_rev_ (mV)**	5 K^+^, 135 Na^+^	−29.1±1.3 (n = 4)	−17.0±1.9* (n = 4)
**P_Na_/P_K_**		0.29±0.02 (n = 4)	0.49±0.04* (n = 4)
**E_rev_ (mV)**	5 K^+^, 135 Li^+^	−74.9±6.7 (n = 5)	−18.9±5.5* (n = 5)
**P_Li_/P_K_**		0.03±0.02 (n = 5)	0.50±0.11* (n = 5)
**E_rev_ (mV)**	5 K^+^, 135 Rb^+^	−18.6±4.4 (n = 4)	−4.1±1.8* (n = 4)
**P_Rb_/P_K_**		0.48±0.09 (n = 4)	0.85±0.06* (n = 4)
**E_rev_ (mV)**	5 K^+^, 135 Cs^+^	−35.6±9.1 (n = 7)	−12.6±0.9* (n = 4)
**P_Cs_/P_K_**		0.31±0.09 (n = 7)	0.59±0.02* (n = 4)
**E_rev_ (mV)**	5 K^+^, 135 NH_4_^+^	−30.5±1.1 (n = 3)	−16.2±3.5* (n = 3)
**P_NH4_/P_K_**		0.27±0.01 (n = 3)	0.52±0.07* (n = 3)
**E_rev_ (mV)**	5 K^+^, 135 MA^+^	−50.0±6.7 (n = 6)	−23.3±1.5* (n = 6)
**P_MA_/P_K_**		0.13±0.05 (n = 6)	0.38±0.02* (n = 6)
**E_rev_ (mV)**	5 K^+^, 135 DMA^+^	−62.5±1.9 (n = 5)	−28.9±1.7* (n = 6)
**P_DMA_/P_K_**		0.05±0.01 (n = 5)	0.29±0.02* (n = 6)
**E_rev_ (mV)**	5 K^+^, 135 TMA^+^	−66.8±1.0 (n = 5)	−50.7±2.8* (n = 5)
**P_TMA_/P_K_**		0.03±0.00 (n = 5)	0.10±0.02* (n = 5)
**E_rev_ (mV)**	5 K^+^, 135 TEA^+^	−83.6±10.0 (n = 4)	−78.2±6.5 (n = 3)
**P_TEA_/P_K_**		0.02±0.02 (n = 4)	0.01±0.01 (n = 3)

Reversal potentials (E_rev_) estimated from leak-subtracted tail currents were used to estimate permeability ratios (P_X_/P_K_) for Na^+^, Li^+^, Rb^+^, Cs^+^, NH_4_^+^, MA^+^, DMA^+^, TMA^+^ and TEA^+^ using the GHK equation (Eq. 1).

* P<0.05 significant difference between C479T and WT HCN4 channels.

**Figure 3:**
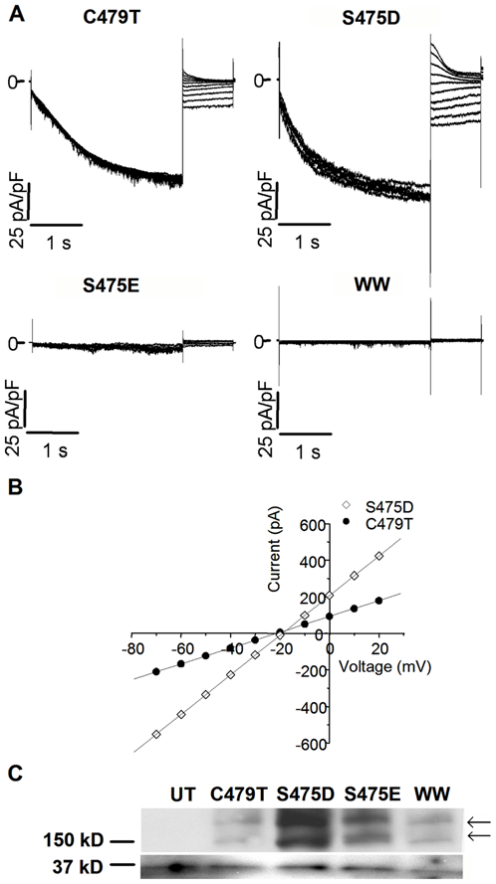
Na^+^ permeability in mutant HCN4 channels. **(A)** Sample traces of C479T, S475D/C479T, S475E/C479T and F471W/K472W/C479T HCN4 currents (labeled S475D, S475E and WW for simplicity) recorded in a 5 mM K^+^/135 mM Na^+^ bath solution, elicited by a 3 s prepulse to −130 mV from a holding potential of 0 mV, followed by a 1 s test pulse between +20 to −70 mV (ΔV = 10 mV). S475E and WW channels did not express currents. **(B)** Representative instantaneous I–V relationships from tail currents of C479T (•) and S475D (◊) channels from recordings performed in a 5 mM K^+^/135 mM Na^+^ bath solution. P_X_/P_K_'s were determined to be −17.0±1.9 (n = 4) and −16.2±2.0 (n = 5) for C479T and S475D channels respectively which were not statistically significant. **(C)** Western blots performed from whole-cell lysates of untransfected (UT) CHO-K1 cells, or cells transfected with C479T, S475D, S475E or WW constructs, with GAPDH used as a loading control (bottom of panel). All mutant channels are highly expressed in both *N*-glycosylated and core bands (←), thus, the lack of current observed from S475E and WW channels is not due to mistrafficking, or low protein production, but rather non-functional protein at the plasma membrane.

In addition to selectivity filter sequence, HCN channels show residue differences in P-loop regions associated with pore rigidity and K^+^ ion selectivity. Specifically based on sequence alignments (Figure 1A), we attempted to restore stabilizing hydrophobic π−bond structures characterized in *KcsA* [15] and *Shaker* channels [23], [24] by replacing F471 and K472 with tryptophans. These mutations were created on a C479T background, since Thr at this position in K^+^ channels plays a role in selectivity [20], [32] by forming the 4^th^ ion binding site [15], [16]. Unfortunately, F471W/K472W/C479T did not yield current in CHO-K1 cells when either 5 mM K^+^/135 mM Na^+^ (Figure 3A) or 140 mM K^+^ was used in the external solution. We also generated S475E/C479T, and S475D/C479T HCN4 channels, in an attempt to introduce a stabilizing salt bridge that has been previously characterized for inward rectifiers [25], [26]. Like F471W/K472W/C479T channels, S475E/C479T HCN4 channels did not generate currents under equivalent conditions (Figure 3A). However, both F471W/K472W/C479T and S475E/C479T HCN4 channels showed high protein expression levels, with both core and *N-*glycosylated bands. Thus, these mutant HCN4 channels traffic properly, despite producing no current, possibly as a result of large voltage shifts in the channel activation. By contrast, S475D/C479T HCN4 channels did generate currents whose permeability ratio for Na^+^ ions (P_Na_/P_K_) in S475D/C479T channels (0.51±0.04), did not differ from C479T channels (P_Na_/P_K_ = 0.49±0.04; P = 0.80) (Figure 3B).

**Figure 4:**
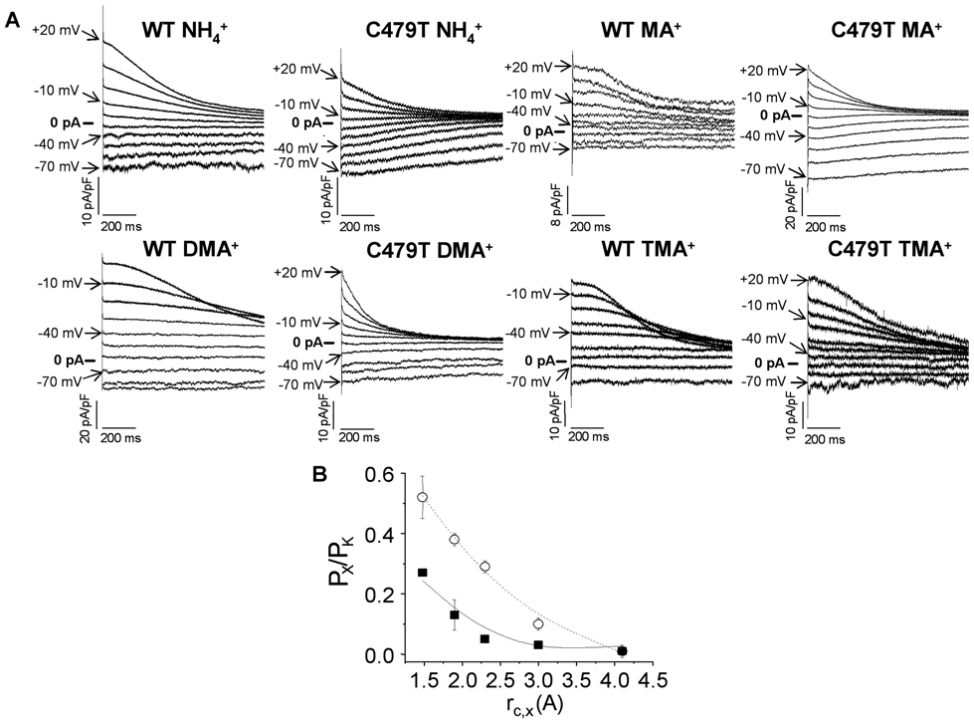
Pore size estimates for WT and C479T HCN4 channels. **(A)** Sample tail currents elicited following an activating “pre-pulse” to −130 mV as shown in Figure 1B for experiments performed in 5 mM K^+^ and 135 mM X^+^ (NH_4_^+^, MA^+^, DMA^+^ and TMA^+^) extracellular bath solution. Using “Exclusive Field Theory” we estimated the minimum pore diameter by plotting P_X_/P_K_ of organic cations NH_4_^+^, MA^+^, DMA^+^, TMA^+^, and TEA^+^ for WT (▪) and C479T (○) channels versus the ionic crystal radius according to Equation (2.15). Fits of these data gave minimum pore diameters of 6.9±0.4 Å (R = 0.93) and 8.7±0.3 Å (R = 0.99) for WT and C479T channels respectively (P<0.05).

We measured the permeability ratios of organic cations of varying size (Figure 4A) which are poorly hydrated and therefore have been used to assess channel pore size. In WT channels, the sequence of P_X_/P_K_ for organic cations was NH_4_^+^ (0.27)>MA^+^ (0.13)>DMA^+^ (0.05)≥TMA^+^ (0.03)≥TEA^+^ (0.02). Although the sequence of organic cation selectivity was unchanged (NH_4_^+^ (0.52)>MA^+^ (0.38)>DMA^+^ (0.29)>TMA^+^ (0.10)>TEA^+^ (0.01)), C479T channels showed large increases in permeability for the cations, MA^+^, DMA^+^ and TMA^+^, compared to WT channels (Table 1). Previous studies have used the “Excluded Field Theory (EFT)” to estimate the pore diameter of various multi-ion channels [28], [33]–[42] from permeation results using the crystal radii of organic cations [43]. The application of EFT to our results (Figure 4B) predicted that the minimum pore radius was larger for WT HCN4 channels (3.5±0.2 Å) than previously estimated for K^+^ channels (∼1.7 Å) [15], [16], [41], [42]. Interestingly, EFT predicted pore radii of 4.4±0.2 Å in C479T channels suggest that replacement of the relatively large cysteine residue of WT HCN4 with the smaller hydroxyl group of threonine enlarges the pore, thereby allowing increased permeability to Li^+^ and Na^+^ ions, as well as large organic cations. Moreover, the selectivity sequence of organic cations (NH_4_^+^ (0.77)>MA^+^ (0.37)>DMA^+^ (0.25)>TMA^+^ (0.06)>TEA^+^ (0.04)) and the estimated minimum pore radius of S475D/C479T channels (4.1±0.3 Å) were unchanged compared to C479T channels (F = 0.23; P = 0.80).

**Figure 5:**
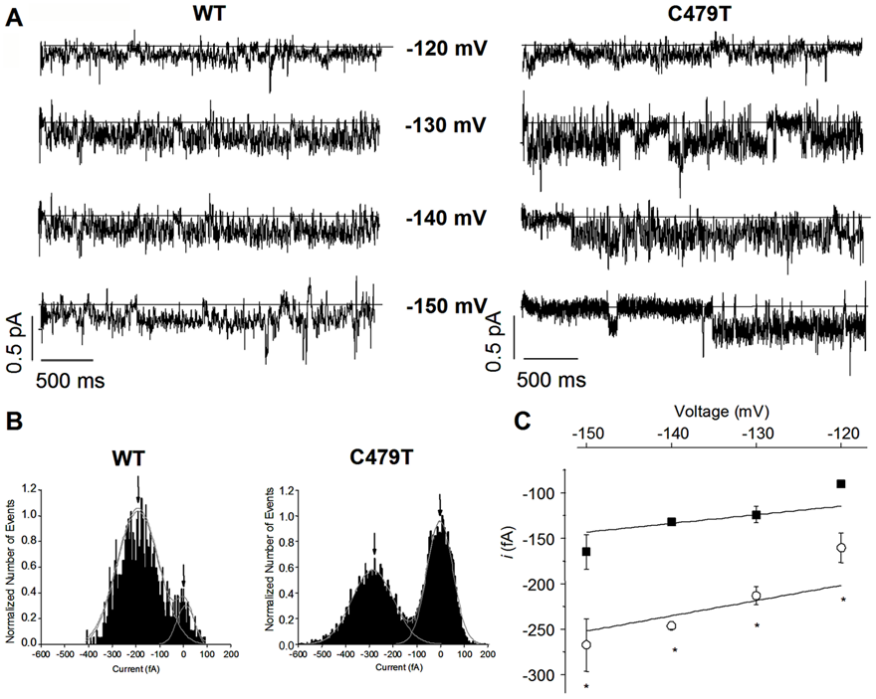
Unitary current (*i*) measurements in WT and C479T HCN4 Channels. **(A)** Single-channel WT and C479T currents recorded at −120, −130, −140 and −150 mV in 140 mM extracellular K^+^. The solid line denotes zero current in the closed channel. **(B)** Sample histograms used to estimate unitary currents (*i*) of WT and C479T channels at −150 mV. Histograms were fit as a dual Gaussian distribution to obtain values for *i*. **(C)** Unitary-current-Voltage (*i*-V) relationship of WT (▪) and C479T (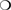) channels between −120 and −150 mV. The unitary conductance (γ), estimated from the slope of a line through the origin, was larger (P<0.05) in C479T channels (1.7±0.1 pS) compared to WT HCN4 (1.0±0.1 pS) (n≥3 for each voltage; *P<0.05).

An increase in pore size for C479T channels compared to WT should be associated with an increased conductance, particularly if permeation and selectivity involves ion sieving [44], as suggested for HCN channels by the monotonic decrease in permeability with organic ion radius (Fig. 4B). To test this conjecture, unitary currents were measured using cell-attached single-channel recordings (Figure 5A) with 140 mM K^+^ in the pipette. Linear regression of the relationship between the unitary current, estimated from frequency histograms of current amplitudes as illustrated in Figure 5B at −150 mV, and the applied voltage allowed estimation of the unitary conductance (γ). For WT HCN4 channels, γ was 1.0±0.1 pS which is similar to previous estimates for native I*_f_* channels in SA node myocytes and pyramidal neuron dendrites (∼1 and ∼0.7 pS) [45]–[47]. By contrast, γ for C479T channels was increased to 1.7±0.1 pS (P<0.05), as expected for channels consistent with an increase in pore diameter compared to WT.

**Figure 6:**
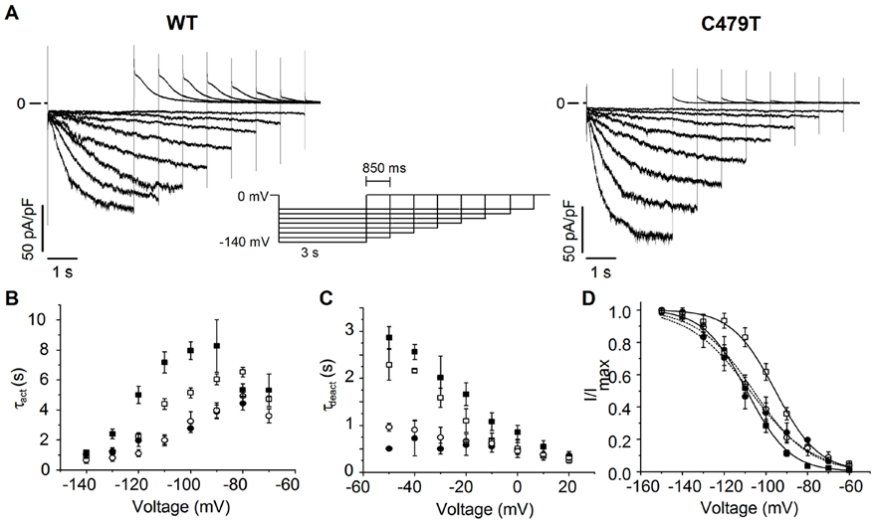
Gating properties of WT and C479T HCN4 channels. **(A)** Representative WT and C479T activation currents elicited by the protocol in the inset. Traces shown were recorded with 5 mM K^+^/135 mM Na^+^ in the extracellular and 140 mM K^+^ intracellular solutions. Similar recordings were performed with 140 mM K^+^ in the extracellular solution. **(B)** Activation time constants (τ_act_) measured using a mono-exponential fit of currents following the initial delay. C479T channels (circles) activated faster* than WT (squares) in 140 mM extracellular K^+^ (filled symbols) (n = 5 and 8 respectively). WT activation kinetics were faster* when most of the extracellular K^+^ was replaced with Na^+^ (5 mM K^+^/135 mM Na^+^; open symbols) (n = 9). C479T activated with the same kinetics following substitution of K^+^ with Na^+^ (n = 5). **(C)** Deactivation time constants (τ_deact_) measured using a mono-exponential fit of currents similar to those in Figure 1 following the initial delay. C479T channels deactivated faster* than WT in both 140 mM K^+^ (n = 5 and n = 7 respectively) and 5 mM K^+^/135 mM Na^+^ (n = 7 and n = 6 respectively) extracelluar conditions. **(D)** Steady-state activation curves from peaks of tail currents elicited at +30 mV following pulses from −150 to −60 mV using a varying pulse duration protocol. WT (▪) channels activated with the same V_1/2_ as C479T (•) channels in 140 mM K^+^ (V_1/2_ = −110±3 mV and −108±4 mV respectively; P = 0.69), however, had steeper* slope factors (*k* = 9.0±0.8 and 12.9±0.6 respectively). Upon ion substitution to 5 mM K^+^/135 mM Na^+^, WT channels (□) activated at a +14 mV* more depolarized potential (V_1/2_ = −96±2 mV) compared to 140 mM K^+^. C479T channels showed no shift in V_1/2_ (−106±3 mV; P = 0.74) in 5 K^+^/135 Na^+^ (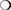) compared to those in 140 K^+^. The slope factor of activation, *k*, of WT channels is unchanged by ion replacement (*k* = 9.2±1.4 in 5 mM K^+^/135 mM Na^+^ respectively; P = 0.57 vs. 140 K^+^), however, remained steeper* than the slope factors in C479T channels (*k* = 11.8±0.8 in 5 K^+^/135 Na^+^; P = 0.34 vs. 140 mM K^+^). P<0.05 where indicated by *.

In addition to changes in permeation, we also routinely observed small, but reproducible differences in gating properties between WT and C479T channels. For example, the time constants of activation (τ_act_) and deactivation (τ_deact_) were smaller (P<0.05) in C479T channels than WT HCN4 channels with 140 mM external K^+^ (Figure 6B). These kinetic changes were also associated with altered steady-state activation properties in C479T channels compared to WT channels. Specifically, the slopes of the steady-state activation curves (*k*) were steeper (P<0.05) in WT channels compared to C479T channels in 140 mM K^+^ (*k* = 9.0±0.8 versus 12.9±0.6 respectively), with no differences (P = 0.69) in the voltage for 50% channel activation (V_1/2_, = −108±4 mV for C479T versus −110±3 mV for WT) (Figure 6D). Furthermore, we also found that replacement of 140 mM external K^+^ with 5 mM K^+^/135 mM Na^+^ with (Figure 6B) accelerated (P<0.05) HCN4 channel activation, without affecting deactivation kinetics (Figure 6C). This replacement also shifted (P<0.05) to more depolarized potentials (V_1/2_ = −96±2 mV) without altering the slope (*k* = 9.2±1.4) (Figure 6D). By contrast, and gating kinetics (Figure 6B and 6C) and steady-state activation properties (Figure 6D) of C479T channels were insensitive to replacement of extracellular K^+^ with Na^+^. This is consistent with previous reports indicating HCN channel activity is dependent on the permeant ion [19], [48], [49]. These effects of C479T replacement on channel gating may originate from a number of mechanisms including changes in pore structure and thereby ion binding, possibly leading to altered pore-gating in selectivity filter as reported previously in K^+^ channels [32], [50], [51].

## Discussion

HCN channels show poor selectivity for K^+^ ion versus Na^+^ ions, despite many structural similarities with K^+^ channels. Since the exquisite selectivity of K^+^ channels for K^+^ has been shown to depend on ‘selectivity sequence’ TT(V/I)GYG as well as a number of other stabilizing interactions involving other P-loop residues it has been suggested that the selectivity properties of HCN channels arise from differences these P-loop regions [1], [13], [14]. Our results demonstrate that L478T/C479T mutant HCN4 channels, which restore the TTIGYG sequence to the pore, failed to produce measurable current despite high levels of of *N*-glycosylated protein expression. Since several K^+^ channels contain leucine or alanine in the equivalent position of L478 (Figure 1A) we went on to examine C479T channels. However, C479T channels exhibited reduced selectivity for K^+^ over Rb^+^, Cs^+^, Na^+^, and especially Li^+^ ions compared to WT HCN4 channels. These results establish that the TIGYG sequence known to be critical for selectivity in K^+^ channels does not confer selectivity to HCN4 channels and indicate that HCN channels may be more structurally divergent from K^+^ than anticipated.

**Figure 7:**
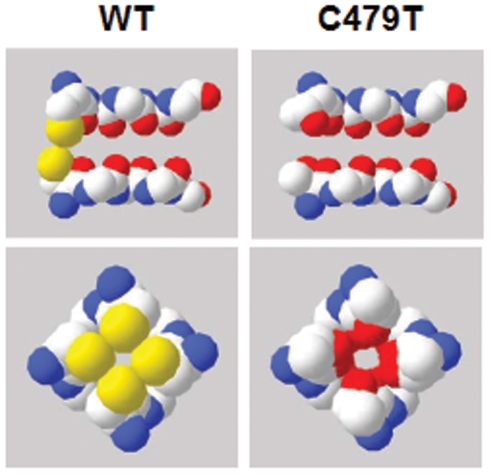
Proposed pore topology in WT and C479T HCN4 channels. A space filled model of the pore sequence in WT and C479T HCN4 channels based on the model of mHCN2 (Giorgetti *et al.*, 2005). The top two panels depict a side view of this sequence in these channels with the intracellular side of the channel on the left. The bottom panels depict a bottom up view of the pore in these channels. The bulk of the −SH group (yellow) in WT HCN4 would limit the pore diameter, and provide a steric barrier thereby limiting ionic permeation and selectivity based on hydrated ionic radius. The C479T mutation increases the pore diameter from 5.4 Å in WT (at the edge of the electron clouds) to 6.2 Å and reduces the steric barrier for ion permeation and ionic selectivity based on effective radius.

Ion selectivity for both WT and C479T HCN4 channels follows a Type IV Eisenman sequence [30], [31], indicating that replacement of C479 with threonine has minimal impact on binding properties of ions in the pore. On the other hand, compared to WT HCN4 channels, C479T channels showed marked elevations in permeability to large organic cations, particularly DMA^+^ and TMA^+^ ions, as well as Li^+^ ions, which have the largest hydrated radius (i.e. 3.8 Å) of the alkali series of cations and are unlikely to be dehydrated due to their very negative free energy of hydration (ΔG_H_ = −120.1 kcal/mol) [52]. These observations suggest that replacement of C479 with threonine enlarged the pore diameter. To further assess and quantify pore size we applied “Excluded Field Theory” (EFT) [43] to our permeation studies. This method was previously applied to accurately predict the dimensions of multi-ion K^+^ channel pores [41], [42] and has been applied to estimate pore sizes for various multi-ion channels [28], [33]–[40]. EFT assumes that ion selection/permeability is dictated primarily sieving mechanisms rather than ion binding properties [43]. The validity of this assumption in HCN4 channels is supported by the monotonic decrease in permeability ratios with increased organic cation diameter (Figure 3B) and by the alkali permeability sequence measured for WT and C479T HCN4 channels which follow an Eisenman Type IV sequences indicative of weak cation binding in the pore. The application of EFT to our organic cation studies predicted that WT HCN4 channels have a pore diameter of ∼6.9 Å in WT HCN4 channels which was increased to ∼8.7 Å in C479T channels. While the basis for this increase in pore diameter remains unclear, it could simply originate from the steric differences between the two residue side-chains. Indeed, assuming the most stable S1-rotamer for the cysteine [53] and threonine side chains in an HCN2 molecular model revealed that the pore diameter increases by ∼1.8 Å (Figure 7) when C479 is replaced by threonine. This is nearly identical to our EFT predictions (∼1.9 Å). Such large pore diameters for WT and C479T HCN4 channels are predicted to readily allow passage of partially hydrated Na^+^ ions in a manner similar to the Na^+^ permeation observed in SK_Ca_, NaK and mutant Kir3.1/3.4 channels [26], [54], [55]. Larger pores can also clearly explain the ∼70% larger single-channel conductance of C479T channels compared to WT HCN4 channels, particularly if permeation in these channels involves a sieving mechanism, as discussed above.

Our estimates of pore size in WT HCN4 channels are clearly larger than K^+^ channel pores (∼3.5 Å) estimated from atomic structures [15], [16] or from EFT [41], [42], and is in good agreement with earlier estimates from I*_h_* channel studies [56] and molecular modeling of spHCN and HCN2 channels [53]. Clearly, the basis for larger pore sizes in HCN4 channels compared to K^+^ channels are unlikely to originate from differences in the amino acid sequences in the selectivity filter. On the other hand, several molecular structures required for K^+^ rigidity and stability in K^+^ channels appear to be absent in HCN4 channels [53]. Moreover, introducing mutions in other regions of the P-loop, linked previously to pore stability in K^+^ channels [15], [23], [25], [26], [54] resulted either in non-functional channels (S475E/C479T and F471W/K472W/C479T) or channels that remained highly permeable to Na^+^ (S475D/C479T). Thus, the underlying architecture of HCN channels appears to be significantly different from K_v_ channels. The molecular basis for these differences in pore size and structure between HCN channels and K^+^ channels will clearly require further studies.

In addition to changes in permeation, we routinely observed reproducible differences in gating properties between C479T and WT HCN4 channels. For example, the C479T replacement resulted in a more shallow voltage-dependence for activation compared with WT HCN4 channels, without affecting the voltage for 50% activation (i.e. V_1/2_). Moreover, ionic substitution had no effect on any gating properties of C479T channels. By contrast, in WT HCN4 channels substitution of extracellular K^+^ with Na^+^ accelerated activation, but not deactivation, rates and induced positive voltage shifts in the steady-state activation. Interestingly, the effects of ion substitution in HCN4 channels differed from that reported previously for HCN1 and HCN2 channels [19], [48], [49]. Clearly, additional studies will be required determine the basis for these differences between HCN4 versus HCN1 and HCN2. Regardless, it is possible that the alterations in gating seen in C479T channels originate from changes in selectivity properties or selectivity filter structure which have previously been shown to modulate “pore-gating” [32], [50], [51].

In conclusion, our data establish that the TIGYG sequence and other pore stabilizing interactions known to be critical for selectivity in K^+^ channels do not confer selectivity to HCN4 channels. Consistent with previous reports, our data suggest that HCN4 channels have much larger pore diameters than K_v_ channels and the C479 residue may form a steric barrier to the permeation of large ions. Overall our data suggests that HCN channels may be less similar in structure to K_v_ channels than originally anticipated.
